# Plasma miR-124 Is a Promising Candidate Biomarker for Human Intracerebral Hemorrhage Stroke

**DOI:** 10.1007/s12035-017-0808-8

**Published:** 2017-11-03

**Authors:** Zifeng Wang, Gang Lu, Johnny Sze, Yao Liu, Sheng Lin, Hong Yao, Ji Zhang, Dan Xie, Quentin Liu, Hsiang-fu Kung, Marie Chia-mi Lin, Wai Sang Poon

**Affiliations:** 1 State Key Laboratory of Oncology in South China, Collaborative Innovation Center of Cancer Medicine, Sun Yat-sen University Cancer Center, Guangzhou, China; 2 Brain Tumor Centre and Division of Neurosurgery, Department of Surgery, Faculty of Medicine, The Chinese University of Hong Kong, Prince of Wales Hospital, Shatin, Hong Kong, China; 3 Laboratory of Medical Genetics, Shenzhen Research Institute of Population and Family Planning, Shenzhen, China; 4 Jiangsu Eng. Laboratory of Cancer Biotherapy, Xuzhou Medical College, Xuzhou, China; 5 Academy of Medical Science, Zhengzhou University, Zhengzhou, Henan China

**Keywords:** Stroke, Intracerebral hemorrhage (ICH), Biomarker, Plasma miRNAs, miR-124

## Abstract

Stroke causes death or long-term disabilities and threatens the general health of the population worldwide. Recent studies have suggested that miRNAs are dysregulated and can be used as biomarkers for diagnosis and prognosis in stroke. The intracerebral hemorrhage (ICH) accounts for 15% of all the stroke cases. However, at present, little is known regarding the functions and clinical implications of miRNAs in ICH. In the present study, we established the collagenase-induced rat ICH model to mimic human ICH syndrome. We profiled the expression of 728 rat miRNAs at different time points in rat brain tissues and plasma post-ICH and identified a set human brain-enriched miRNAs that had changed expression level in the plasma of rat ICH. Among them, the expression levels of miR-124 displayed significantly synchronous alterations in rat plasma and brain tissue during ICH progression. They were significantly elevated at the acute injury phase (day 1 and 2), gradually decreased during the delayed recovery phase (day 7, 14 and 30), and finally restored to normal levels at late recovery phase (day 60). We further determined the plasma expression profile of miR-124 from human ICH patients. Similar to the pattern observed in rat ICH model, our results indicated that immediately after patients reached the hospital, the average plasma concentrations of miR-124 increased more than 100-fold in 24 h, then decreased gradually on day 2, 7, 14 and to near normal level on day 30. Taken together, these results strongly suggested that plasma concentration of miR-124 is a promising candidate biomarker for the early detection and predictive prognosis of human ICH.

## Introduction

Stroke is a cerebrovascular disease and the second leading cause of death, accounting for 9.6% of the total mortality worldwide [1]. About 80% of stroke cases are due to cerebral ischemia (ischemic stroke), and 15% of stroke cases are caused by intracerebral hemorrhage (hemorrhagic stroke, ICH) [2, 3]. ICH is resulted from ruptured blood vessel(s) in the brain leading to the pooling of blood in the brain parenchyma and the formation of hematoma [4]. These processes are associated with multiple biological processes including oxidative damage, inflammation, edema formation, neuronal cell death, and dysregulation of multiple genes [5–7]. The mortality of ICH is often as high as 50% within the first month after ICH, with half of the deaths occurring within 48 h [8]. Timely diagnosis and treatment are important for ICH patients.

MicroRNAs (miRNAs) are small non-coding RNA molecules (19–30 nt) that are endogenous regulators of gene expression [9]. Many miRNAs are expressed in a tissue- and/or cell-specific manner and their expression patterns are reflective of underlying patho-physiologic processes. miRNAs can be detected in serum and plasma in a remarkably stable form, making them desired biomarkers for human diseases [10]. Circulating miRNAs, including miR-1, miR-122, miR-124, miR-133a, miR-192, and miR-208, have been proved to be sensitive and specific markers for monitoring acute tissue injuries [11]. It has been shown that miRNAs are temporally regulated during progression/reperfusion of cerebral ischemia, miR-138 and miR-145 in total blood could be used as diagnostic markers [12]. Similarly, in traumatic brain injury (murine model), correlations have been found between the temporal miRNA expression profile and several biological processes underlying the brain injury [13]. In human patients, Tan et al. has demonstrated that blood miRNA from young ischemic stroke patients (18 to 49 years) could be used to identify the disease progression and the stroke subtype [14]. Recently, miRNA-210 has been shown to be a novel blood biomarker in acute cerebral ischemia [15].

As compared to ischemic stroke, little study related to the role of miRNAs in ICH has been reported. Recently, *Liu* et al. examined the brain and blood miRNA expression profile changes using a blood infusion (lysed blood, fresh blood, or thrombin) ICH rat model at 24 h after injury. They found that several miRNAs changed more than 2-fold in both brain and blood, suggesting the possibility of blood miRNAs as ICH biomarkers [16]. To date, the function and clinical implications of miRNA in human ICH patient are not well understood. In the present study, we established the collagenase-induced rat ICH model to mimic human ICH syndrome and then measured 728 different miRNAs expressed in plasma samples and collagenase-damaged brain tissues at different time points. We further identified the brain-specific miR-124 as a promising candidate biomarker in ICH patient plasma for early detection and of intracerebral hemorrhage.

## Materials and Methods

### The Rat Collagenase-Induced ICH Model

All procedures involving animals were performed to minimize pain or discomfort in accordance with current protocols approved by the Animal Research Ethics Committee of The Chinese University of Hong Kong. A cohort of 21 male Sprague-Dawley rats with a mean age of 12 weeks and body weight of 220 g were used.

Subject rats were randomly separated into two groups, one injected with PBS (sham surgery control, *n* = 3) and the other injected with collagenase (ICH model, *n* = 18). These rats were first anesthetized by intraperitoneal injection of 30 mg/kg ketamine (Alfasan, FarmaVet SA, Bucharest, Romania) and 4 mg/kg xylazine (Alfasan) then fixed to a stereotactic frame (David Kopf Instruments, Tujunga, CA). A surgical incision was made in the midline of the skull. Using a microdrill, a puncture was made at 0.2 mm posterior to the bregma and 3 mm left lateral to the midline. A Hamilton syringe with a 26-gauge needle was inserted 6 mm below the skull surface, and 1.5 μL of PBS or 0.21 collagenase digestive units (Sigma) was slowly injected into the internal capsule over 5 min. The needle was allowed to remain in place for a further 5 min before gentle withdrawal. The burr hole was sealed with bone wax, and the skin incision was sutured. Rats were allowed access to food and water ad libitum in isolator cages at 25 °C under a 12-h light/dark cycle. This was basically a collagenase-induced ICH into the striatum based on Rosenberg et al. [17] and MacLellan et al. [18]. The sham surgery group was anesthetized and sacrificed at 1 day post-PBS injection, while the ICH model groups were operated at days 1, 2, 7, 14, 30, and 60 post-collagenase injection (*n* = 3 at each time point). The brain hematoma and the cardiac blood were harvested, and the blood was further separated into blood cells and plasma fractions.

### Experimental Design

**Fig. 1:**
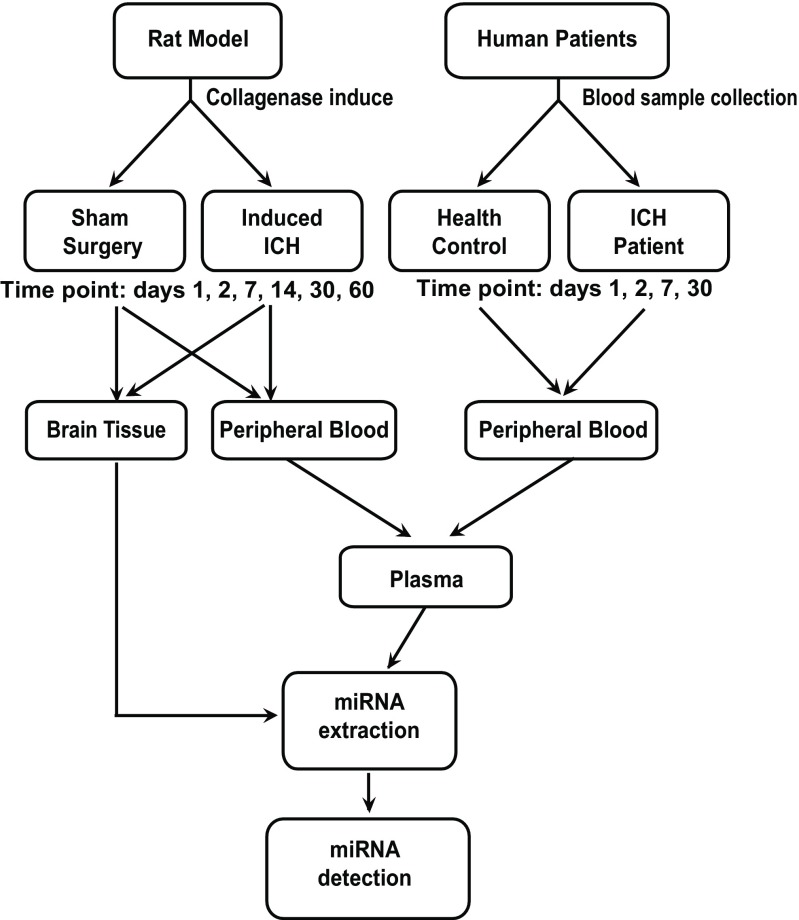
Flow diagrams showing experimental design of this study. Collagenase-induced preclinical rat model of ICH was first established and evaluated. Brain tissue and plasma miRNAs were extracted and measured and then confirmed in human patient plasma

The experimental design of this study is summarized in Fig. 1. For the rat ICH studies (Fig. 1 left panel), we first established the rat model of collagenase-induced ICH, which is believed to mimic human ICH syndrome [17, 18]. Then, the plasma and brain tissue samples were collected, with the sham surgery group at day 1 (*n* = 3) and the ICH model groups (*n* = 18) at days 1, 2, 7, 14, 30, and 60 (*n* = 3 per time point) post-collagenase injection. For human ICH studies, as shown in Fig. 1 right panel, 20 patients who were diagnosed as ICH and hospitalized in the Prince of Wales Hospital in The Chinese University of Hong were recruited in this study. The first blood samples were collected within 24 h after the admission, and then on day 2, 7, and 30. EDTA blood was centrifuged at 1600 g for 10 min at 4 °C, and plasma was transferred to new tubes followed by further centrifugation at 16000 g for 10 min at 4 °C. Then, the supernatants were collected into fresh tubes and stored at – 20 °C until ready for experiments.

### Plasma miRNA Extraction from Human ICH Patients and Rat ICH Models

The human and rat plasma miRNAs were extracted according to the procedure of EKO Ng et al. [19]. Briefly, total RNA containing small RNA was extracted from 500 μL of plasma using Trizol LS reagent (Invitrogen, CA, USA) and miRNeasy Mini Kit (Qiagen, Hilden, Germany) according to the manufacturer’s protocol with the following modifications: Trizol LS reagent was added to plasma samples in volumetric ratio about 4:3. After phase separation by chloroform addition and centrifugation, 1.5 volumes of 100% ethanol was added to the aqueous phase, and the mixture was loaded into miRNeasy column according to the manufacturer’s instructions. A supplement of the bacteriophage MS2 (Roche) was added to increase the yield of total RNA extracted.

### miRNA Extraction from Rat Brain Tissues

For miRNA extraction from brain tissues (hippocampus), the total RNA was extracted using Trizol (Invitrogen) in accordance with the manufacturer’s instructions. The final elution volume was 30 μL and the concentrations of all RNA samples was quantified by NanoDrop 1000 (NanoDrop, Wilmington, Delaware, USA). RNA samples were stored at − 80 °C before use.

### miRNA Profiling by Locked Nucleic Acid (LNA) qPCR Assay

To achieve high sensitivity and specificity profiling of miRNAs in less abundant medium, cDNA synthesis and quantitative real-time PCR (qPCR) were performed using the miRCURY LNA™ Universal RT microRNA PCR system (Exiqon, Denmark) according to the manufacturers’ instructions. In brief, miRNA samples from the same group (*n* = 3) were pooled together for miRNA microarray profiling. The RNA was tailed with a poly(A) sequence at their 3´end and then reverse transcribed into cDNA using a universal poly(T) primer with a 3´end degenerate anchor and a 5´end universal tag.

The qPCR screen of the miRNAome was performed with microRNA ready-to-use PCR mouse and rat panel I + II V2 R with primers for 752 rodent miRNAs (728 of these are present in rat, Exiqon, Denmark). The reactions were performed on an ABI 7900 thermocycler (Applied Biosciences, 10 min at 95 °C with 45 amplification cycles at 95 °C 10 s, 60 °C 1 min) according to the manufacturer’s instructions. The qPCR data were analyzed using the 2^-ΔCt^ quantitative method, where ΔCt = Ct target − Ct global mean. Expression intensities were log2 transformed and median-centered by subtracting the median value of each array from each intensity value. Hierarchical clustering was performed with complete linkage and Euclidean distance measurements with HCE 3.5 software [20].

Technical confirmation of the candidate miRNAs from the miRNAome screen was carried out in triplicate using the same RNA template and primers as for the screen. The primers are based on Exiqon’s patented LNA™ technology, and all primers were commercially obtained from Exiqon, Denmark. The reactions were performed in a 96-well plate format (Pick-&-Mix panel from Exiqon) on a Roche Lightcycler 480 according to the manufacturer’s instructions.

### Prediction of miRNAs Target Genes and Biologic Function

We predicted miRNA target genes by publicly available software TargetScan 5.2 web tool. Then, the candidate targets were further submitted to DAVID algorithm to analyze the pathways involved.

### Statistics Analysis

The data were analyzed by unpaired two-tailed Student’s *t* test and expressed as mean ± standard deviation (SD). Differences were considered statistically significant at *p* value < 0.05.

## Results

### Identification of miRNAs with Altered Expression Levels During Rat ICH Progression

The miRNA profiling was conducted by locked nucleic acid (LNA) qPCR assay as described in Methods. To achieve high sensitivity and specificity for miRNA profiling, the miRCURY LNA™ Universal RT microRNA PCR was performed. A total of 742 miRNAs were analyzed by the mouse and rat panel-based real-time PCR and LNA-enhanced miRNA-specific primers. To reduce the endogenous errors of high throughput analysis, we first measured the miRNA profiles of the pooled samples (*n* = 3) in each experimental group, and the mean values of each group were used for further analysis.

**Fig. 2:**
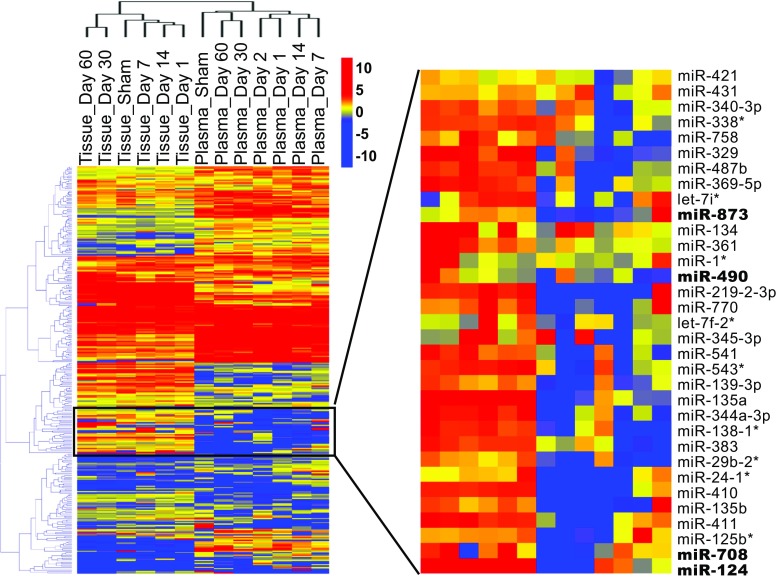
miRNA expression profile in rat brain tissue and plasma after ICH. Hierarchical cluster analysis of miRNAs expression profile in brain tissue and plasma of rats subjected to ICH for day 1, 2, 7, 14, 30, 60, or to sham surgery. The later time points luster together with sham surgery and away from the early time points (left panel). The miRNAs which were relatively high expressed in brain tissue and significantly changed expression in plasma were displayed in right panel. Red indicates high expression and blue low expression

Of the 748 mature *Rattus norvegicus* (rno)-miRNAs analyzed by the panel, 301 miRNAs were detected in our profiling analysis. Hierarchical clustering analysis showed that samples from brain tissues and plasma, respectively, clustered together (Fig. 2, left panel). Furthermore, the later time points (day 30 and 60 at the recovery phase) clustered together with sham surgery and away from the early time points. In addition, we identified 33 brain-specific (enriched) miRNAs with significantly changed expression levels in the plasma (Fig. 2, right panel).

### Identification of Human Brain-Specific miRNAs, miR-124, miR-490, miR-873, and miR-708 with Changed Expression Level in the Plasma of Rat ICH

An ideal plasma biomarker of brain injury should be abundantly and preferentially produced in the brain, while not present or at low concentrations in the blood and other body fluids. Upon brain injury, such biomarkers are expected to be released into the systemic circulation or other body fluid, where they can be detected in a blood-based assay or assay of another accessible body fluid [11]. From this perspective, the brain specifically enriched miRNA(s) are the best candidate(s) for ICH. By data mining, we first search the miRNAs which are brain-specific and enriched but also weekly expressed in the blood. Pablo Landgraf et al. have detected the expression of more than 600 miRNAs in 147 human tissues and cell lines by next-generation sequencing [21]. We analyzed the expression levels of these miRNAs in the brain and hematopoietic organs. The top 20 human brain-enriched miRNAs were listed as shown in Table 1.

**Table 1:** Brain-specific miRNAs and their expression in hematopoietic organs

miRNA	Total copies	Brain		Hematopoietic organs		Hematopoietic/brain ratio
		Copies	Percentage (%)	Copies	Percentage (%)	
hsa-miR-124	1613	1568	97.21	14	0.87	0.0089
hsa-miR-490	16	14	87.50	0	0.00	0.0000
hsa-miR-488	53	46	86.79	0	0.00	0.0000
hsa-miR-873	15	12	80.00	0	0.00	0.0000
hsa-miR-218	66	40	60.61	0	0.00	0.0000
hsa-miR-138	79	46	58.23	15	18.99	0.3261
hsa-miR-153	84	48	57.14	24	28.57	0.5000
hsa-miR-129	52	29	55.77	14	26.92	0.4828
hsa-miR-379	103	51	49.51	7	6.80	0.1373
hsa-miR-136	167	82	49.10	7	4.19	0.0854
hsa-miR-338	27	13	48.15	1	3.70	0.0769
hsa-miR-204	23	11	47.83	2	8.70	0.1818
hsa-miR-708	153	67	43.79	20	13.07	0.2985
hsa-miR-128	407	173	42.51	182	44.72	1.0520
hsa-miR-139	27	11	40.74	8	29.63	0.7273
hsa-miR-99a	904	336.5	37.22	165	18.25	0.4903
hsa-miR-92b	71	26	36.62	6.5	9.15	0.2500
hsa-miR-877	40	14	35.00	6	15.00	0.4286
hsa-miR-125b	1317	456	34.62	214	16.25	0.4693
hsa-miR-137	148	50	33.78	0	0.00	0.0000

The copy number of miRNAs is based on 147 human cells and tissues detected by Pablo Landgraf et al. 2007

**Fig. 3:**
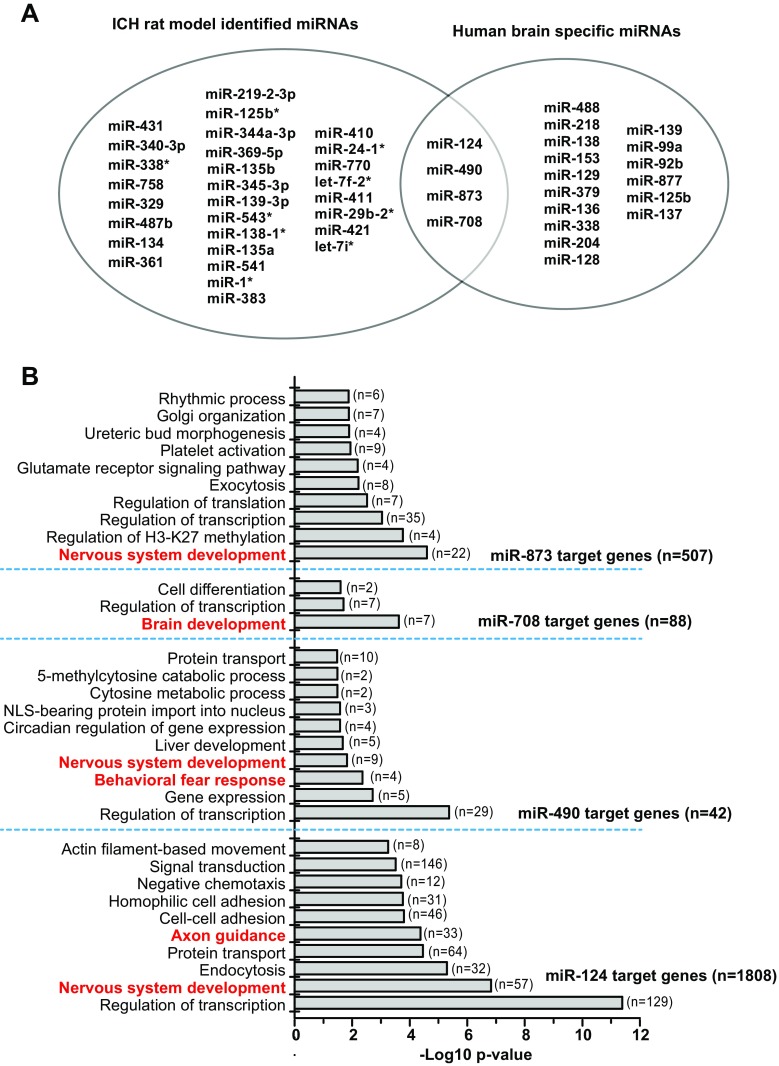
miR-124, miR-490, miR-873, and miR-708 are human brain-specific miRNAs and change expression in ICH rat plasma. **a** Venn diagram with ICH rat model-identified miRNAs and human brain-specific miRNAs. **b** Target genes of miR-124, miR-490, miR-873, and miR-708 were predicted by TargetScan, respectively. Then, the predicted target genes were submitted into DAVID to analyze the biological process (BP) using Gene Ontology. “*n*” indicates the gene number in each GO term

Then, we examined the expression levels of these 20 human brain-enriched miRNAs in our rat ICH model screening. As shown in Fig. 3a, miR-124, miR-490, miR-873, and miR-708 are human brain-specific miRNAs with changed expression in the plasma of rat ICH. Since miRNAs exert their function through suppressing gene translation, we predicted these four altered miRNAs by TargetScan 7.0 web tool, respectively. Then, the predicted targets were submitted into DAVID to analyze the enriched biological processes. Many bioprocess concerns in the brain/nervous system development were identified as the top-ranked (Fig. 3b), suggesting that these miRNAs were brain-derived.

**Fig. 4:**
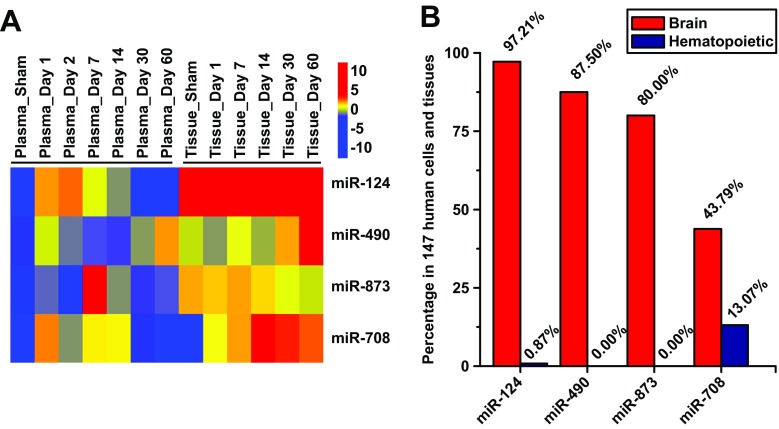
Expression of selected miRNA in ICH rat model and human. **a** miR-124, miR-490, miR-873, and miR-708 expression patterns in the ICH rat brain and plasma. **b** miR-124, miR-490, miR-873, and miR-708 proportion in the human brain and hematopoietic organ relative to 147 human cells and tissues

The expression patterns of these four miRNAs in the plasma and brain tissues during ICH progression were shown in Fig. 4a. Among these miRNAs, the expression of miR-124, miR-490, and miR-873 expressed lower than 1% in hematopoietic organs. Among them, miR-124 is most highly expressed (more than 97%) in the brain (Fig. 4b). Hence, we chose miR-124 as the candidate miRNA for further study.

### Brain-Specific/Enriched miR-124 Was Expressed Synchronously in Plasma and Brain Tissue in the Rat ICH Stroke Model

To comprehensively evaluate the expression pattern of miR-124 in ICH, we monitored the expression of miR-124 in both brain tissue and plasma in rat ICH stroke model at six time points, which include acute injury phase (day 1 and day 2), delayed recovery phase (day 7 and day 14), and recovery phase (day 30 and day 60). We first used relative qPCR to detect miR-124 expression in ICH rat brain tissues and plasma. Rno-miR-21 and rno-miR-23b were used as the endogenous control as suggested by the “Norm-finder” algorithm, a model-based variance estimation predicting the stability of a given miRNA across samples. The qPCR data for the technical confirmation was analyzed using the 2–ΔΔCt quantitative method.

**Fig. 5:**
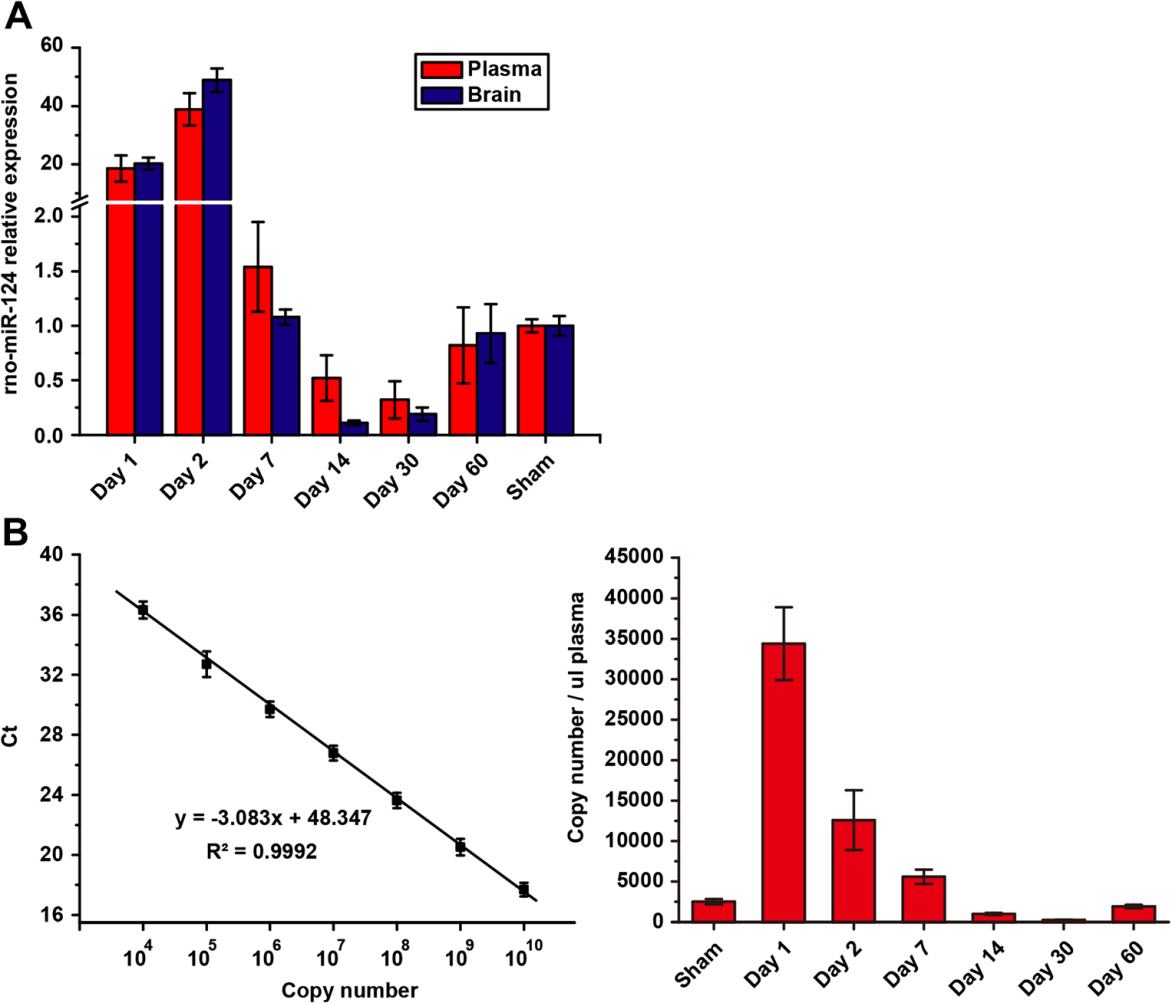
miR-124 expressed synchronously in plasma and brain tissue in the rat ICH model. **a** Relative plasma and tissue miR-124 expression was measured by relative qPCR. Results are presented as group mean (SD) for the -fold change normalized to the miR-21 and miR-23b expression at a given time point. **b** Plasma miR-124 expression was measured by absolute qPCR. Left panel, standard curve generated by SYBR Green real-time PCR. Serially diluted standard commercial mature miR-124 was amplified. Threshold cycle (Ct) values were plotted against copy number. Right panel, detection of miR-124 in human ICH patient plasma samples after the admission to the hospital

As shown in Fig. 5a, the expression levels of miR-124 in both brain tissue and plasma were synchronous and significantly elevated at the acute injury phase (day 1 and day 2), gradually decreased during the delayed recovery phase (day 7 and day 14) and recovery phase (day 30). Notably, on day 14 and 30, the expression levels of miR-124 in both brain tissue and plasma decreased to levels significantly below the normal level (sham group). At later recovery phase (day 60), the expression levels of miR-124 in the brain and plasma returned to normal level.

To further confirm the result, we determined plasma miR-124 level using absolute qPCR. As shown in Fig. 5b, using absolute qPCR, similar expression patterns were observed.

### The Plasma of Human ICH Patient Samples Exhibited Similar Expression Patterns as Compared to Rat ICH

**Fig. 6:**
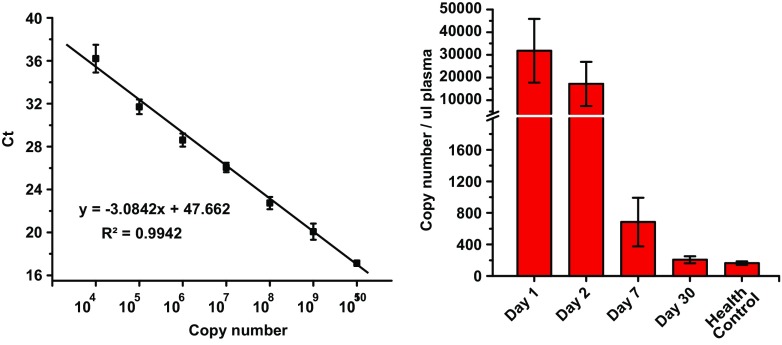
miR-124 expressed in human ICH patient plasma samples after the admission to the hospital. Plasma miR-124 expression was measured by absolute qPCR. Left panel, standard curve generated by SYBR Green real-time PCR. Serially diluted standard commercial mature miR-124 was amplified. Threshold cycle (Ct) values were plotted against copy number. Right panel, detection of miR-124 in human ICH patient plasma samples after the admission to the hospital

To our knowledge, miRNA expression pattern in human ICH patient plasma samples has not been reported. Here, we measured the expression pattern of miR-124 in plasma samples from 20 human ICH patients admitted in the Prince of Wales Hospital (Table 2). As shown in Fig. 6, the relative miR-124 expression level was highest on day 1 (*n* = 20, 31,765 ± 14,049 copies per ul plasma) within 24 h after patient admission to the hospital, and gradually decreased starting from day 2 (*n* = 20, 17,176 ± 9739 copies per ul plasma) and day 7 (*n* = 16, 684 ± 308 copies per ul plasma), day 30 (*n* = 6, 206 ± 45 copies per ul plasma), as compared to normal healthy individuals (*n* = 6, 163 ± 23). These findings are consistent with the results obtained from the rat ICH preclinical animal study. These results suggested that plasma concentration of miR-124 is a promising candidate biomarker for possible early detection, diagnosis, and prognosis of human ICH.

**Table 2:** Clinical information of health control and ICH patients

	Health control	ICH patients
	*(n = 6)*	*(n = 20)*
Mean age	58.67 ± 7.97	59.75 ± 9.53
Male	3 (50.0%)	13 (65.0%)
Hypertension	1 (16.7%)	13 (65.0%)
Smoker	1 (16.7%)	12 (60.0%)
Diabetics	0 (0.00%)	5 (25.0%)
Dyslipidemia	1 (16.7%)	6 (30.0%)
Previous stroke	0 (0.0%)	2 (10.0%)

## Discussion

Currently, two prevalent animal models were used to mimic human ICH syndrome, the collagenase-induced [17] and blood infusion models [22]. However, there are significant differences between these two models. Our present study selected the collagenase-induced ICH rat model because collagenase-induced ICH in rat has a high mortality which is age-dependent with marked brain edema, like human ICH. This model has been suggested to better mimic human ICH syndrome [17, 18]. A comparison study [18] has shown that despite similar initial hematoma volumes, collagenase-induced ICH resulted in a greater blood-brain barrier breakdown and more damage to the striatum, substantia nigra, white matter, and cortex. Tissue loss continued over 4 weeks in the collagenase model. In contrast, in the blood model, MRI revealed faster hematoma resolution, and little increase in the volume of tissue lost from 1 to 6 weeks. Moreover, functional deficits recovered more quickly and completely. Generally, the blood model mimics a single large bleed that was thought to occur in most ICH patients, but does not reproduce spontaneous bleeding. However, more recent clinical data suggest that bleeding continues for up to 24 h in many ICH patients. The rapid and apparently complete behavioral recovery in the blood model is at odds with the persistent and debilitating deficits seen in ICH patients. Furthermore, a rapid functional recovery is a significant problem in studies that seek to assess long-term outcome. Even though collagenase does not discriminate among different brain tissues, it produces massive bleeding and mortality, similar to that in human ICH. This model has been used extensively in investigations of ICH [23–30], including studies on the long-term deficits after collagenase-induced ICH [31].

Our results also suggest that plasma miRNAs are desirable biomarkers for ICH. At present, the diagnosis and prognosis of stroke rely on imaging techniques such as magnetic resonance imaging. Biomarkers sampled from blood are particularly desirable [32]. An accurate and reliable blood biomarker for diagnosis and risk prediction of stroke is currently lacking. Several protein biomarkers have been suggested to be elevated on cerebral infarction. However, these markers are neither detected in the early phase of stroke nor are specific for brain injury. Ideal biomarkers should be easily accessible, reliable, cost-effective, and non-invasive. As small and stable molecules, the plasma miRNAs are attractive biomarkers for human diseases. However, the expression pattern, function, and application of miRNAs in human ICH are not well understood.

The blood biomarkers were believed to be released into the circulation after injury [11]. However, the existence of blood-brain barrier which strictly controls the exchanges between the blood and the brain makes the stroke biomarkers in blood difficult. The candidate macromolecule protein biomarkers of stroke were believed to depend on the disruption of the blood-brain barrier [33], which limit their application. Therefore, identification of biomarkers that could cross blood-brain barrier is highly desirable. Although the ability of 21 nt small molecule miRNA to cross the blood-brain barrier remains inconclusive, results from many published results including our present study suggest that they are likely able to cross the barrier.

In addition, it has been demonstrated that blood miRNAs remain stable upon exposure to severe conditions that induce almost immediate degradation of free RNA, such as boiling, very low or high pH, and extended storage [34, 35]. The expression levels are reproducible and indicative of the disease state [36]. Blood miRNAs consistent among healthy individuals and can be detected in both plasma and serum [37]. It has been reported that plasma miRNAs are sensitive and specific biomarkers for various tissue injuries [11] and pathological conditions [11, 38–40].

Consistently, we used the collagenase-induced rat ICH model and evaluated the expression patterns of 728 rat miRNAs at different time points in rat brain tissues and plasma post-ICH. Results from this study revealed that a brain specific-enriched miRNA, miR-124, was expressed synchronously in brain tissue and plasma in the rat ICH stroke model. We further evaluated miR-124 expression pattern in the plasma samples of human ICH patient and showed that miR-124 exhibited similar expression pattern. Taken together, results from this study strongly suggest that changes of plasma concentration of miR-124 are promising biomarkers for early detection, diagnosis, and prognosis of human ICH.

Previously, miR-124 has been suggested as a potential biomarker in tissue injury [11, 41] and cerebral infarction [31]. In this study, in the rat collagenase-induced ICH model and the plasma of human ICH patients, we showed that miR-124 is significantly up-regulated during acute injury phase, suggesting that it is derived from the injury of the brain. The plasma and brain expression levels were then decreased and even reduced on delayed recovery phase, suggesting the healing of the brain injury. Finally, it was returned to normal expression level when the rats were fully recovered.

The miR-124 is the most abundant miRNA in the brain. Due to its extraordinary enrichment in metazoan nervous systems through more than 500 Ma of evolution, miR-124 is one of the best-studied miRNAs in various organisms, by both basic biologists interested in molecular mechanisms of miRNA regulation and neuroscientists investigating brain development and function. As predicted by TargetScan and analyzed by DAVID, the target genes (*n* = 1808) of miR-124 mainly function in the regulation of transcription (*n* = 129) and nervous system development (*n* = 57) (Fig. 3b). These results suggest that miRNA-124 works through regulating the transcription of multiple pathways depending on the change of micro-environments. In addition, the top-ranking target gene is argonaute RISC catalytic component 2 (AGO2), which is related to cell stress. A highly significant correlation between AGO2 expression and cellular growth rate (*p* < 0.05) has been reported [42]. Therefore, it is possible that elevated miR-124 during early injury will markedly suppress AGO2 to inhibit cellular growth and direct cells to wound healing, while in the later recovery phase, reduced mi/r-124 could increase AGO2 to promote cell growth.

The possible clinical implications of plasma miR-124 pattern and concentrations in the detection, diagnosis, and prognosis of human ICH patients remain to be elucidated using larger number of patient samples. These include the possible correlation between brain edema size, the potential associations between the miRNA profiles and the gene expression profiles, as well as ICH grade and clinical outcome. In addition, the functions and mechanisms of miR-124 in ICH require further investigation to determine whether miR-124 could be used as a potential therapeutic target. Knowledge gained is especially important when investigating the nature of ICH pathogenesis. In addition, miRNAs involved in stroke may provide novel therapeutic targets for ICH.
